# In Silico Evaluation of Natural Flavonoids as a Potential Inhibitor of Coronavirus Disease

**DOI:** 10.3390/molecules27196374

**Published:** 2022-09-27

**Authors:** Piyush Kashyap, Mamta Thakur, Nidhi Singh, Deep Shikha, Shiv Kumar, Poonam Baniwal, Yogender Singh Yadav, Minaxi Sharma, Kandi Sridhar, Baskaran Stephen Inbaraj

**Affiliations:** 1 Department of Food Technology and Nutrition, School of Agriculture, Lovely Professional University, Phagwara 144401, India; 2 Department of Food Technology, School of Sciences, ITM University, Gwalior 474001, India; 3 Centre of Bioinformatics, University of Allahabad, Prayraj 211002, India; 4 Department of Food Technology, Bhai Gurdas Institute of Engineering and Technology, Sangrur 148001, India; 5 MMICT & BM (HM), Maharishi Markandeshwar Deemed to be University, Mullana, Ambala 133207, India; 6 Department of Quality Control, Food Corporation of India, New Delhi 110001, India; 7 Department of Dairy Engineering, College of Dairy Science and Technology, Lala Lajpat Rai University of Veterinary and Animal Sciences, Hisar 125004, India; 8 Laboratoire de Chimieverte et Produits Biobasés, Département AgroBioscience et Chimie, Haute Ecole Provinciale du Hainaut-Condorcet, 11, 7800 ATH Rue de la Sucrerie, Belgium; 9 UMR1253, Science et Technologie du Lait et de l’œuf, INRAE, L’InstitutAgro, Rennes-Angers, 65 Rue de Saint Brieuc, F-35042 Rennes, France; 10 Department of Food Science, Fu Jen Catholic University, New Taipei City 242 05, Taiwan

**Keywords:** flavonoids, COVID-19, in silico studies, M^pro^, ACE2

## Abstract

The recent coronavirus disease (COVID-19) outbreak in Wuhan, China, has led to millions of infections and the death of approximately one million people. No targeted therapeutics are currently available, and only a few efficient treatment options are accessible. Many researchers are investigating active compounds from natural plant sources that may inhibit COVID-19 proliferation. Flavonoids are generally present in our diet, as well as traditional medicines and are effective against various diseases. Thus, here, we reviewed the potential of flavonoids against crucial proteins involved in the coronavirus infectious cycle. The fundamentals of coronaviruses, the structures of SARS-CoV-2, and the mechanism of its entry into the host’s body have also been discussed. In silico studies have been successfully employed to study the interaction of flavonoids against COVID-19 M^pro^, spike protein PL^pro^, and other interactive sites for its possible inhibition. Recent studies showed that many flavonoids such as hesperidin, amentoflavone, rutin, diosmin, apiin, and many other flavonoids have a higher affinity with M^pro^ and lower binding energy than currently used drugs such as hydroxylchloroquine, nelfinavir, ritonavir, and lopinavir. Thus, these compounds can be developed as specific therapeutic agents against COVID-19, but need further in vitro and in vivo studies to validate these compounds and pave the way for drug discovery.

## 1. Introduction

**Figure 1:**
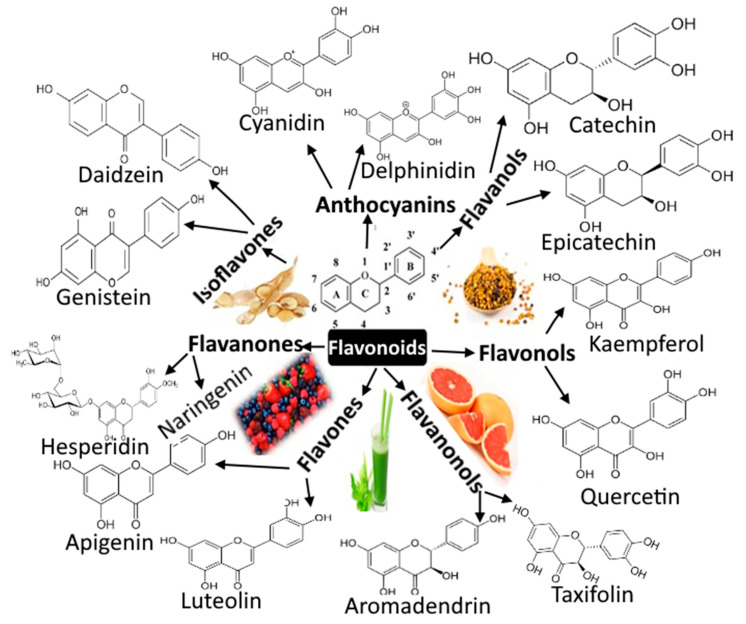
Flavonoids’ basic structure and various flavonoids involved in counteracting coronavirus.

Coronavirus disease (COVID-19), an infectious disease caused by the newly discovered coronavirus (severe acute respiratory syndrome coronavirus-2 (SARS-CoV-2)), first emerged in December 2019 in Wuhan in China and spread to almost all countries, infecting approximately 500,186,525 people by13 April 2022 [1]. Due to its rapid spread, COVID-19 was considered a pandemic disease by the World Health Organization (WHO). Currently, several medicines from Pfizer, Moderna, Covishield, Covaxin, Astrazeneca, Sputnik, and many others are used universally for COVID-19 cure [2]. Plenty of other trials are currently ongoing in different countries with some positive outcomes. The scientists and researchers are endeavoring to discover a novel vaccine or develop novel approaches from available anti-viral drugs that will be a promising solution against this pandemic [3,4]. In this stressful period, in silico docking studies seem to be a propitious tool for drug discovery and development in which the ligand (drug) molecules interact with the target protein (receptor) binding sites [5]. Thus, the in silico-based studies are of keen interest to researchers to identify potential hit molecules, screen the active sites virtually, optimize the lead compounds, etc., while developing the drugs [6,7,8,9]. Xu et al. [10] recommended nelfinavir as a potential inhibitor of SARS-CoV-2 after examining 1903 approved drugs using homology modelling, molecular docking, and binding free energy calculation. Flavonoids are a class of polyphenols present in many fruits, vegetables, and seeds. These are hydroxylated phenolic molecules, which consist of two benzene rings (A and B rings) linked by a heterocyclic pyrene ring (C ring) (Figure 1). These flavonoids can be interesting options as natural inhibitors against SARS-CoV-2, as they lack systemic toxicity and are considered pleiotropic compounds, i.e., the functional groups of these compounds might interact with various cellular targets, blocking several pathways. They can also synergize with different conventional drugs [11,12]. 

Most existing drugs inhibit proteases, and some flavonoid compounds are known to prevent the chymotrypsin-like protease (3CL) [13,14,15,16,17]. The polyphenolic extract of plants such as *Isatis indigotica*, *Torreya nucifera*, and *Houttuynia cordata* showed the prevention ofthe 3CL protease enzyme [18,19,20]. Some recent studies also showed the inhibitory potential of flavonoids such asapigenin-7-glucoside, catechin, epicatechin-gallate, luteolin-7-glucoside, kaempferol, naringenin, and quercetin against SARS-CoV-2 [21]. Khaerunnisa et al. [22] recommended diosmin and hesperidin as major flavonoids to fight against COVID-19 after docking 1500 drugs using the SARS-CoV3-chymotrypsin-like protease (3CLpro) (PDB: 2DUC), whereas diosmin was ranked 22 among 4600 drugs based on the lower binding energy compared to 97% of the top 30 antivirals, as well as the formation of more hydrogen bonds with the active site [23]. In another study, eighty flavonoids were evaluated for molecular dynamics docking with the SARS-CoV-2 protease 3CLpro (PDB: GLU7). The top three candidates for docking at the active site were hesperidin, rutin, and diosmin [24]. Some natural compounds such as baicalin, scutellarin, hesperetin, nicotianamine, and glycyrrhizin have been predicted to have the ability to bind the ACE2 receptor with the potential for anti-SARS-CoV-2 action. Furthermore, quercetin, daidzein, puerarin, epigallocatechin, epigallocatechingallate, gallocatechingallate, and kaempferol have been shown to suppress SARS-CoV 3CL proteolytic activity [25,26,27].

Thus, several recent studies examined the inhibitory effects of flavonoids against SARS-CoV-2; however, such investigations have not been compiled and presented in a single frame yet to provide the impact of flavonoids in combating COVID-19 [27,28,29,30,31]. Therefore, the present review was written to discuss the potential of flavonoids against COVID-19 so that researchers can access all data comprehensively related to this. This review also covers the fundamentals of coronaviruses, the structures of SARS-CoV-2, and the mechanism of its entry into the host’s body. However, major emphasis is given to the recent in silico-based molecular docking and simulation studies reporting flavonoids as potential inhibitors of SARS-CoV-2.

## 2. An Overview and Structure of COVID-19/SARS-CoV-2

According to the International Committee on Taxonomy of Viruses (ICTV), coronaviruses (CoVs) belong to the family Coronaviridae, sub-order Cornidovirineae, and order Nidovirales under Riboviria [32,33]. There are four classes of CoVs: (i) Alpha-CoV, (ii) Beta-CoV, (iii) Gamma-CoV, (iv) and Delta-CoV, out of which Alpha-CoV (human CoV NL63, porcine transmissible gastroenteritis CoV, porcine respiratory CoV, etc.) and Beta-CoV (bat CoV HKU4, severe acute respiratory syndrome (SARS)-CoV, Middle East respiratory syndrome (MERS)-CoV, mouse hepatitis CoV, etc.) infect mammals, Gamma-CoV (infectious bronchitis CoV) may contaminate avian species, and Delta-CoV (porcine Delta-CoV) can infect mammalian, as well as avian species [34,35,36]. These are followed by seven coronaviruses that may infect human hosts: 229E (Alpha-CoV), HKU1 (Beta-CoV), MERS-CoV (Beta-CoV), NL63 (Alpha-CoV), OC43 (Beta-CoV), SARS-CoV (Beta-CoV), and 2019-novel CoV (Beta-CoV). These are responsible for affecting the respiratory tract, resulting in flu-like symptoms (common cold, fever, and cough). Other symptoms include watery diarrhea, bronchiolitis, rhinitis, and sinusitis [37,38]. Among these, the most pathogenic strains are MERS-CoV, SARS-CoV, and 2019-novel CoV, while the remaining strains cause mild respiratory diseases in humans [36]. On 11 February 2020, ICTV announced another name of 2019-novel CoV as “severe acute respiratory syndrome coronavirus 2 (SARS-CoV-2)” because it contains similar types of receptors, mainly the receptor-binding domain (RBD) and receptor binding motif (RBM) in the viral genome [39,40,41,42]. As per the Research Collaborator for Structural Bioinformatics (RCSB) database, the sequence of 2019-novel CoV or SARS-CoV-2 comprises a single PDB (PDB ID: 6LU7) in complex with N3 (inhibitor) [43]. On the basis of genomic comparison, SARS-CoV-2 resembles (96%) *Rhinolophus affinis* bat the most and showed 80% similarity to *Rhinolophus sinicus* bat [44]. Another study reported the 99% genomic resemblance of novel coronavirus with pangolins and proposed the animal as an intermediate host to the virus [45]. The genome of SARS-CoV-2 is highly vulnerable to numerous recombination processes, thus producing new strains with different degrees of virulence [46].

**Figure 2:**
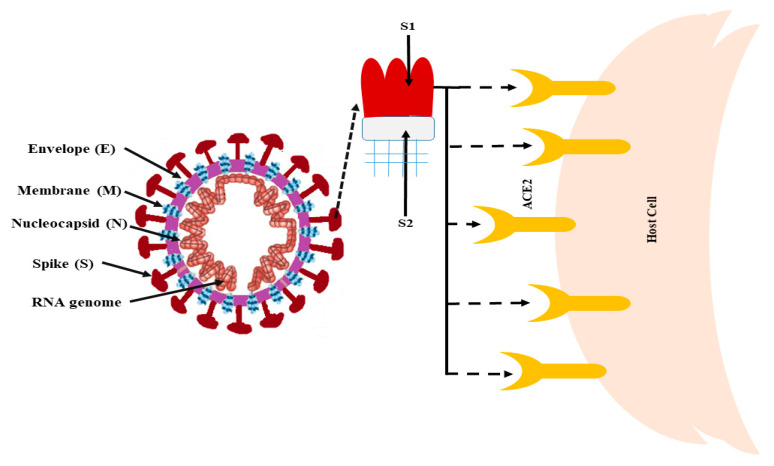
Basic structure of SARS-CoV-2 and its interaction with the host cell. ACE2, angiotensin-converting enzyme 2. Figure 2 is available under an open-access Creative Commons Attribution License (CC-BY).

The structures of coronaviruses are huge, pleomorphic, but typically spherical, non-segmented, positive-stranded RNA viruses, i.e., +ssRNA having a 5’-cap structure and 3’-poly-A tail, and possess the largest genome (27–32 kb) of all RNA viruses [47]. The CoV genome contains seven genes, conserved in the following order: ORF1a, ORF1b, S, OEF3, E, M, and N in the 5’ to 3’ direction. The genome is mostly (2/3rd) surrounded by ORF1a/b, which further generates the two viral replicase proteins: polyproteins (PP) 1a and PP1ab [48,49]. These PPs, when processed, produce the 16 mature non-structural proteins (NSPs), which participate in several viral functions, and some encode the mRNA to synthesize structural proteins [48]. The major structural proteins of SARS-CoV-2 are: (i) envelope (E), (ii) membrane (M), (iii) nucleocapsid (N), and (iv) spike (S) (trimeric) [47,50]. They contain protrusions (80–120 nm diameter) of glycoproteins above the surface [51]. Besides these, some coronaviruses may also encode the envelope-linked hemagglutinin-esterase protein (HE) [52]. The nucleocapsid protein (N) creates a helical capsid that contains the RNA genome inside it. The genome is further covered by an envelope that possesses the remaining three proteins [50,51,52,53]. The M and E proteins are important in viral assembly, while the S-protein is responsible for the virus’s giant crown-like projections above the surface. Therefore, this virus was named coronavirus (in Greek, corona (κορώνα) means crown) [54,55]. The S-protein connects to the cell membranes of the host through these protrusions by targeting the angiotensin-converting enzyme 2 (ACE2) receptors, which are mostly present in the respiratory epithelium and alveoli of lungs [56,57]. The ACE2 protein is also expressed in various human organs, notably the kidney and gut, making them CoV’s primary targets [58]. This virus also contains a particular amino acid residue (Gln493) in the receptor binding motif (RBM), which assists the S-protein in attaching ACE2 proteins [59]. Figure 2 shows the structure of SARS-CoV-2, indicating the binding of the S-protein to the host cell. Furthermore, S-proteins are also required for defining viral host range and tissue tropism. In addition, they are the primary inducers of immunological responses in the host [60]. 

**Figure 3:**
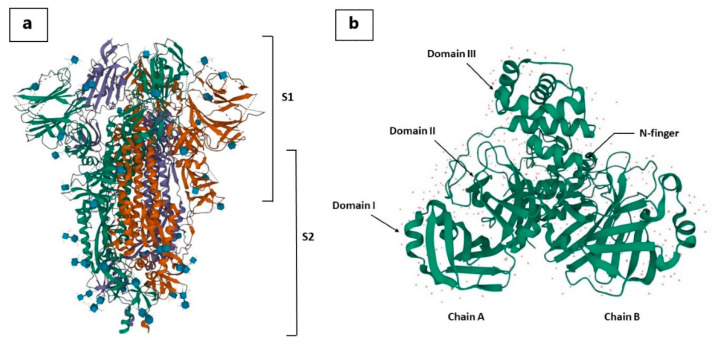
**The** 3D structure of the spike protein (**a**) and 3CL protease (**b**) of SARS-CoV-2.

There are the following major sections of the S-protein: (i) ectodomain, (ii) single-pass trans-membrane anchor, and (iii) intracellular tail, which are important for anchoring the host cells [61]. The ectodomain comprises two subunits: (i) S1 receptor-binding and (ii) S2 membrane fusion subunit, which have a clove-trimeric or crown structure [62,63]. The S-protein is cleaved into the S1 and S2 domains by the proteases (Figure 3), inducing a conformational alteration, which activates S2, followed by the insertion of the fusion peptide (FP) into the membrane for fusion, which allows the virus to enter the cell [41]. The S1subunit consists of the N-terminal domain and receptor-binding domain, whereas the fusion peptide, heptapeptide repeat sequence, and cytoplasm domain are present in the S2 subunit. After binding, the S-protein faces structural changes for entering the host cell [36,64]. Therefore, the best way to combat COVID-19 is to neutralize SARS-CoV-2 from entering the host cells, which has been seen for previous viruses of its class [60,65,66].

Another important part of SARS-CoV-2 is a non-structural protein, i.e., 3C-like protease (3CL pro). 3CLpro is a three-domain cysteine protease with a highly conserved active site across all coronaviruses (Figure 3). The first two domains are six-stranded β-barrels, and in the cleft of these domains, there is a substrate binding site. The second domain (Domain II) is linked to the C-terminal domain (Domain III) by a long loop. Domain III is a global cluster of five helices and involved in 3CL pro proteolytic activity [61]. The substrate binding site is located in the cleft between Domain I and Domain II, which binds through N-terminus residues, which are located between Domain II and Domain III with roles in the formation of the substrate binding site.

## 3. Potential Approaches to Control SARS-CoV-2 Infection

The outbreak of SARS-CoV-2 poses a high risk due to the human-to-human transmission, making COVID-19 a public health emergency of international concern. To combat the spread of the virus, many preventative measures such as hand washing, maintaining cleanliness, social distancing, isolation, mask usage, keeping a strong immunity, and travel restrictions are being followed.

**Figure 4:**
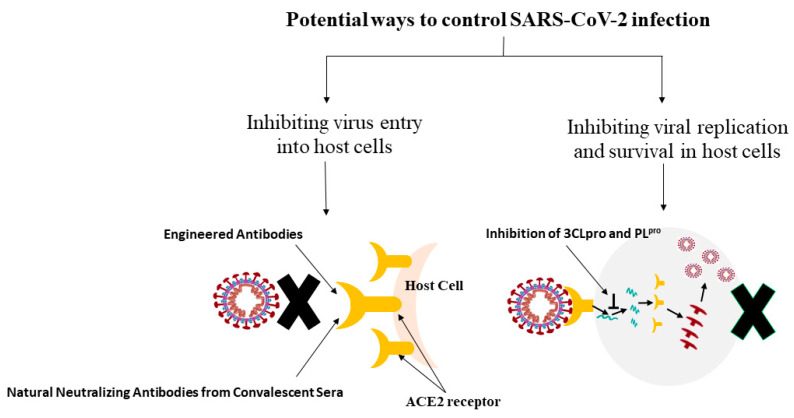
Summary of potential approaches to control SARS-CoV-2 infection.

The few potential technical approaches to control SARS-CoV-2 infection are summarized in Figure 4 and discussed below.

### 3.1. Inhibiting Virus Entry into Host Cells

This is the best approach to stop COVID-19infection, which can be accomplished by preventing the binding of the spike protein of the virus to the host cell’s ACE2 receptor [16]. This can be achieved by using natural neutralizing antibodies from convalescent sera (serum obtained from recovered persons) and engineered antibodies. Such antibodies can be found in several forms such as a single-chain variable section attached to ACE2. These prevent the access to the soluble receptor binding domain of SARS-CoV-2, which inhibits ACE2. This further restricts the access of the virus and soluble ACE2 binding to SAR-CoV-2 to sequester it competitively and remove it from the cell-surface-bound ACE2 in host cells [67,68]. Further, the fragment crystallizable (Fc) region of natural and engineered antibodies may exclude the virus via phagocytosis and immune activation [69]. A study showed the inhibition of S-protein binding to ACE2 by emodin and promazine [70]. The smaller molecule drugs from drug repurposing approaches can also attach to the S-protein, thus interrupting the interaction with ACE2 [71,72]. Besides these, the protease inhibitors are reported to be beneficial in preventing virus entry because of S-protein priming by trans-membrane protease serine (TMPRSS2) [16,73]. S-protein priming includes the cleavage of the S-protein by cellular proteases such as cathepsins, TMPRSS2, and furin to fuse the viral and cellular membranes. Further, the potential flavonoid molecules may also bind the active sites of the virus and inhibit its entry into the host cell. This approach will be discussed in detail in Section 4 and Section 5.

### 3.2. Inhibiting the Viral Replication and Survival in Host Cells

The virus, if it enters, can be blocked by inhibiting the major proteases such as3CLpro and PL^pro^ due to their significant role in the proteolysis of the viral polyprotein into the functional moiety [47]. Several flavonoids, cinanserin, diarylheptanoids, and nelfinavir, have been shown to prevent the 3CLpro or PL^pro^, making them potential agents to stop the replication of SARS-CoV-2 [15,74,75]. Moreover, the host cells produce type-1 interferons such as IFNα and IFNβ when responding to viral infection. They break down the viral RNA and inhibit the synthesis and assembly of the viral protein [76]. The researchers must explore the type-1 interferons used for decades to manage viral infection by blocking virus replication [77]. Neuraminidase inhibitors such as oseltamivir and zanamivir can inhibit virus’s replication, budding, and infection [78,79]. Scientists must study the synergistic effects of these options for managing COVID-19.

## 4. Flavonoids and Their Effect on SARS-CoV

**Figure 5:**
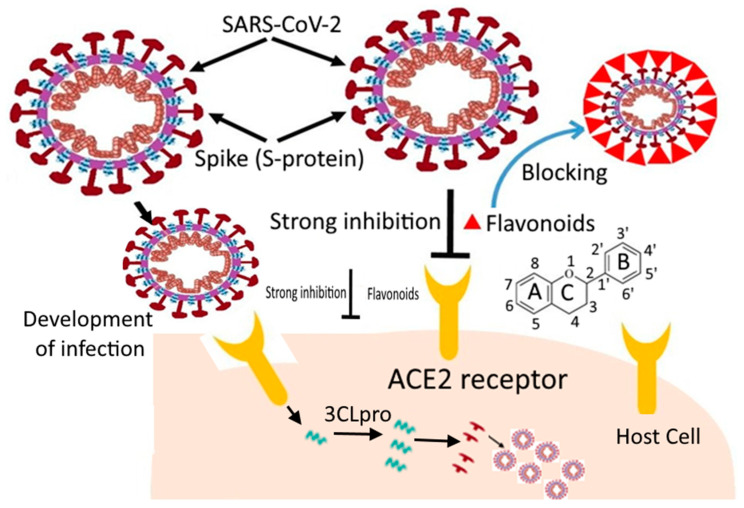
Systematic representation of the mechanism of flavonoid inhibition against SARS-CoV-2. Figure 4 is available under an open-access Creative Commons Attribution License (CC-BY).

Many herbal extracts and their derivatives have been used in traditional medicine to treat various diseases, including viral infections [80]. The positive effects of natural plant-derived flavonoids on neurodegenerative disorders [81], type-2 diabetes [82], atherosclerosis [83], cardiovascular diseases [84,85], and cancer [85,86,87,88] are well known. A broad variety of antiviral effects of flavonoids have also been reported for many viruses, such as polio, astrovirus, respiratory syncytial virus, type 3 parainfluenza virus (PIV-3), and type A influenza virus (Flu A) [89,90,91,92]. Flavonoids are important natural products that belong to plant secondary metabolites and are widely present in fruits and vegetables. They have also been studied against various DNA and RNA viruses [93]. The inhibitory action of flavonoids against COVID-19is shown in Figure 5. Different flavonoids such as quercetin, puerarin, apigenin, daidzein, amentoflavone, luteolin, epigallocatechin, and many others have been shown to suppress SARS-CoV 3CLproproteolytic activity. 3CL pro is highly conserved among human coronavirus and is a very favorable drug target. After coronavirus infection, the translation and activation of 3CLpro occurs, which cleaves the pp1a and pp1ab proteins into other non-structural proteins, necessary for the replication of the virus. Various molecular docking studies revealed that flavonoids inhibit the activity of 3CL pro up to 80% and further replication of coronavirus [25]. As a result, the antiviral impact of flavonoids is expected to specifically suppress SARS-CoV 3CL proactivity [15].

The potential antiviral effect of flavonoids has also been shown against SARS-CoV and MERS-CoV. Biaclin, flavones, and glycoside were isolated from *Scutellaria baicalensis* screened for fRhK4 cell lines derived from 10 SARS-CoV-affected patients, and after 48 h, no significant cytotoxicity was observed with an EC_50_ value of 12.5–25 µg/mL. However, the peak serum concentration of biaclin reached up to 74 µg/mL after 360 mg was administered intravenously to the patients [94]. Similarly, the dose-dependent inhibition of luteolin and quercetin was also observed against SARS-CoV infection with EC50 values of 10.6 µM and 83.4 µM, respectively [95]. Polyphenols from *Brussonetia papyrifera* were tested for their ability to inhibit the proteolytic activity of 3CLpro and PL^pro^ proteases from SARS-CoV and MERS-CoV. Papyriflavonol, anaquercetin derivative, showed potent inhibitory action against the PL^pro^ protease of SARS-CoV with an IC_50_ value of 3.7 µM. The rest of the compounds showed a dose-dependent inhibition with higher IC_50_ values [96]. In another study against MERS-CoV 3CLpro, 40 flavonoids were evaluated at a 20 µM concentration, and four flavonoids, namely isobavachalcone, quercetin-3-β-D-glucoside, herbacetin, and helichrysetin, were found to be most effective with an IC_50_ value of 33.85, 37.03, 40.59, and 67.04 µM, respectively. Flavonoids interact with the S1 and S2 sites of the 3CLpro protease, according to a molecular docking investigation. The hydrogen bonding formed between the 4-hydroxyl group of helichrysetin and the hydroxyl group of Tyr54 of MERS-CoV protease showed the better affinity of helichrysetin because Tyr54 is located deep inside the hydrophobic S2 site [97].

An attractive approach was proposed to screen the possible inhibitors of SARS-CoV N protein by mimicking on a glass chip, which includes the direct attachment of viral RNA to the N protein. The investigation demonstrated that only (−)-catechingallate and (−)-gallocatechin-3-gallate could remove the binding of N protein to the RNA oligonucleotide among the 23 polyphenolic compounds. The binding affinity on the built biochip was decreased in compounds following a concentration-dependent approach from 0.005 μg/ml. They revealed a 40% inhibition potential at a concentration of 0.05 μg/mL [98]. Therefore, such experiments showed the potential effect of flavonoids against SARS-CoV, which would support humanity against theCOVID-19 pandemic.

### In Silico Studies of Flavonoids against SARS-CoV-2

In computer-aided drug design, molecular docking (MD) plays an essential role in analyzing various binding interactions of the potential drug with distinct domains and active sites of the targeted molecules to find the lead compound [99]. Several types of research revealed that, by in silico study, some herbal plant (harsingar, aloe vera, and giloy) extracts inhibit SARS-CoV-2’s main protease activity. 

The amino acid sequences of 3CLpro of SARS-CoV and SARS-CoV-2 are nearly identical, with a 96% distinction and 99% similarity [100]. 3CLpro is among the few proteins produced in infected cells and cleaves more significant proteins into smaller ones, which enable the cell to infect other cells [101]. Therefore, in order to avoid viral replication, the aim is to identify the drug or compounds that bind to the surface of 3CLpro in the particular pocket or active site, which is also used by COVID-19 to generate numerous copies of itself in infected host cells. Emphasis is given to discovering and developing new inhibitory agents againstSARS-CoV-2 proteases, for which flavonoids have triggered scientists’ attention as potential anti-SARS-CoV infection agents. However, some flavonoids such as baicalin, scutellarin, and hesperetin exhibit an anti-SARS-CoV-2 effect. These flavonoids can bind with ACE2 and block the entry of SARS-CoV-2 into the host cells. Several flavonoids have an antagonistic impact on SARS-CoV-2. Apigenin, daidzein, luteolin, amentoflavone, epigallocatechin, gallocatechingallate, quercetin, and kaempferol were primarily studied for the inhibition of the proteolytic action of SARS-CoV 3CLpro [20,102,103]. 

In contrast with the actions of peptide-derived inhibitors, the inhibition action of flavonoids against SARS-CoV cysteine proteases was higher in the low micro-range. The selectivity of flavonoids was due to their structural features, exposing the link between two-phenyl groups and inhibitory capability [96]. This is also demonstrated by the IC_50_ values of quercetin (IC_50_ = 8.1 µM), amentoflavone (IC_50_ = 8.3 µM), and quercetin-3-β-galactoside (IC_50_ = 42.79 µM) [20,94]. Molecular docking of all flavonoid structures was performed against the active region of the M^pro^ protein. The docking study showed a number of configurations, which were graded in order to determine the optimum binding modes. The three compounds with the highest active site affinity are quercetin 3-rhamonoside, myricetin 3-rutinos, and myricetin 3-rutinos [104]. Docking studies were conducted to estimate the binding affinity of flavonoids, which showed that the proteolytic action of SARS-CoV 3CLproand the spike protein could be inhibited by apigenin, luteolin, quercetin, daidzein, epi-gallocatechin, and kaempferol [15].

An in-silico study reported that quercetin, hispidulin, and cirsimaritin have better inhibition than hydroxyl-chloroquine against SARS-CoV-2 [105]. The studies of natural flavonoids in the active sites of the SARS-CoV-2 main protease show binding scores of −8.007 for quercetin. The binding sites of the SARS-CoV-2 main protease show effective interaction and hydrogen bond formation with HIS163, HIS164, CYS145, HIS41, and GLU166 [106]. Pandey et al. [107] reported that by molecular docking, flavanoid compounds had better inhibitory action against the spike protein when screened using the AutoDock and PatchDock tools than standard drugs. Therefore, baicalin exhibited the best inhibitory action against the SARS-CoV-2 spike protein. Gogoi et al. [108] reported the docking of 44 flavonoids against SARS-CoV-2 M^pro^, wherein the non-toxic compound was subjected to MD simulation process and 3D-QSAR predicted the IC_50_.It was observed that five compounds among 44 flavanoids had allow binding energy in the docking process with SARS-CoV-2 M^pro^. This compound formed an H-bond with the HIS41 and CYS145 residues with SARS-CoV-2 M^pro^. Gogoi et al. [108] therefore suggested taxifolin as the best inhibitor against SARS-CoV-2 M^pro^. Bhardwaj et al. [109] reported the molecular docking analysis of bioactive molecules of tea extract against SARS-CoV-2. It was profound that molecule oolonghomobisflavan-A, theasinensin-D, and theaflavin-3-*O*-gallate had higher docking scores than the repurposed drugs. These molecules were then subjected to MD simulation at 100 ns, and the results were validated using MM-PBSA binding free energy calculation. Oolonghomobisflavan-A showed a greater number of H-bonds and the least binding energy. Therefore, this molecule was considered as a potential inhibitor of SARS-CoV-2. 

Pandey et al. [99] showed that fisetin combined with SER 730, THR 778, and HIS 1058 residues via H-bonding had hydrophobic association with the S2-domain residues: ILE 870, PRO 880, and THR 732.In comparison, quercetin made H-bonds with LYS 733, LEU 861, MT 731, SER 730, PRO 1057, GLY 1059, HIS 1058, and ALA 1056 and had hydrophobic interaction with ILE 870, ASP 867, MET 730, VAL 860, and PRO 863.However, quercetin’s interaction was controlled by H-bonding by the involvement of an additional 5-OH group in the chromone ring rather than a comparable alternative for the S2 domain. The same situation occurred when they were involved in interactions with various S2 domain residues. In addition, quercetins and vitamin C together had a synergistic antiviral impact because their antiviral and immune-modulating effects overlap and because ascorbate has the ability to recycle quercetin, increasing its effectiveness [100]. Such affordable and safe treatments must be validated before they can be used in the current global health crisis.

In a study, the flavonoid quercetin with a docking score of −10.90kcal/mol was recorded as a potential candidate. Quercetin interacted with eight potent H-bond interactions with amino acid residues LYS5, ALA7, GLN127, LYS137, and GLU290 against SARS-CoV-2 M^pro^ [110]. Cherrak et al. [104] performed a molecular dynamics (MD) simulation on the M^pro^ of SARS-CoV-2 with a selected inhibitor. Swain et al. [111] investigated the docked complex with the highest score performed by using a GROningen Machine for Chemical Simulations (GROMACS) for 100 ns to observe the stability of the selected complex [112]. The parameters and ligand topologies were generated using the PRODRG server (http://prodrg1.dyndns.org/submit.html). Native protein and effective docking complexes with the SPC-E water model were simulated. The water molecule was added to the protein, and the docking complex system was neutralized by adding Na+ ions and energy minimization of the docking complex using the 50,000 steepest descent steps. After minimizing the complex system, equilibrium was observed in twosteps: number of particles, volume, and temperature (NVT) equilibration was performed for 2 ns, and number of particles, pressure, and temperature (NPT) equilibration was performed for 10 ns. The final step in the MD simulation was performed for the protein system for a 100 ns time scale. In the MD simulation, the root-mean-squared deviation (RMSD) plot was used to observe the stability of the complex and the root-mean-squared fluctuation (RMSF) graph was used to monitor the flexibility of the structure. The protein and ligand binding free energy were calculated by combining the molecular mechanic/Poisson–Boltzmann surface area (MM-PBSA) with MD. 

**Figure 6:**
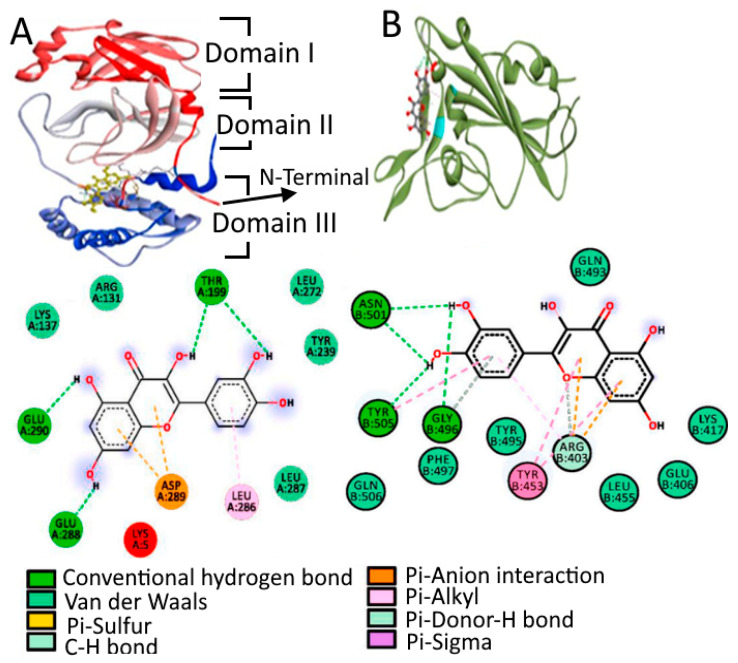
(**A**) Binding mode of M^pro^ with quercetin via amino acid interactions and (**B**) binding mode of spike protein and quercetin via amino acid interactions. Adapted with permission from Vijai kumar et al. [113].

In another study, kaemferol, quercetin, and fisetin bind to the hACE2-S-protein complex through MD simulations on hACE2 and S-protein binding interfaces. This was also confirmed using molecular mechanic/Poisson–Boltzmann surface area (MM-PBSA) analysis, which reported the minimum binding free energy ΔGbind of −22.17 ± 3.04 kcal/mol for quercetin, which suggests the highest binding affinity for the hACE2-S-protein. Kaemferol showed the lowest binding affinity (ΔGbind= −15.07 ± 2.42 kcal/mol), whereas the binding energy ΔGbind of −21.11 ± 3.49 kcal/mol was shown for fisetinto bind hACE2-S-protein. This implies that the compatibility of quercetin and fisetin with hACE2-S is approximately the same, which is in line with docking findings, showing likewise a docked value ΔGbind = −8.50 Kcal/mol. Likewise, quercetin was an attractive option out of 60 distinct compounds that interacted with the SARS-CoV-2 M^pro^ and spike protein in another report. It interacts with M^pro^ at Glu290 and Asp289 with a −9.2 kcal/mol binding affinity, while the spike protein was firmly bound to Gly496, Asn501, Tyr505, and Tyr453, with a −7.8 kcal/mol binding affinity, as seen in Figure 6 [113]. Ngwa et al. [114] also examined hesperetin (−9.1 kcal/mol), myricetin (−8.9 kcal/mol), and novel flavonoids such as Linebacker (−9.2 kcal/mol) and caflanone (FBL-03G) (−7.9 kcal/mol), which exhibited strong binding affinity to the ACE2 receptor’s spike protein, helicase, and protease sites. These flavonoids could bind as well or better than chloroquine (−4.1 kcal/mol). Other flavonoid molecules such as procyanidin b2 and Mangiferin also exhibited a higher binding affinity with the M^pro^ as compared to previously suggested COVID-19 inhibitors such asramdesivir, avipiravir, and hydroxychloriquinone [115].

COVID-19 3CLpro inhibitory compounds were investigated using the proteolytic technique by Jo et al. [15]. Flavonoids that inhibit SARS-CoV 3CLpro were discovered by utilizing a synthetic peptide labelled with Edans-Dabcylfluorescence resonance energy transfer (FRET). The inhibitory effect of all the compounds in the assay was tested at 20 µM. In the flavonoid’s library, three flavonoids, i.e., herbacetin, rhoifolin, and pectolinanrin, were effective at inhibiting SARS-CoV 3CLpro enzyme activity by reducing the fluorescence intensity. The IC_50_ value of herbacetin, rhoifolin, and pectolinanrin was calculated by the dose-dependent inhibitory curve and found to be 33.17 µM, 27.45 µM, and 37.78 µM, respectively. Docking studies indicated that the S1 and S2 sites are involved in the binding of herbacetin due to the extra 8-OH group, which was predicted to develop increased binding affinity at these sites. On the other hand, rhoifolin and pectolinanrin also showed higher binding affinity around the S1 and S2 sites, but the carbohydrate groups of these glycosylated flavonoids are responsible for this affinity. Rhoifolin’s higher binding affinity might be due to coordinated binding across the S1, S2, and S3 sites.

*Salvadora persica* flavonoids also showed the potential of inhibiting COVID-19 protease through a docking study. The aqueous extract from the aerial parts of plants, i.e., stem and leaves, was subjected to LC-HRESIMS for metabolic profiling, and 11 flavonol glycosides were found. All the flavonoids showed almost the same binding affinity asN3, which fits inside the substrate-binding pocket of SARS-CoV-2 M^pro^. Only isorhamnetin-3-*O*-glycoside showed less affinity than the SARS-CoV-2 M^pro^ inhibitor duranavir. Isorhamnetin-3-*O*-rutinoside andkaempferol-3-*O*-rutinoside showed the maximum binding affinity due to disaccharide rutinooside (α-L-rhamnopyranosyl (1-6)-β-d-glucopyranose) at the third position, which is essential for its activity, whereas isorhamnetin-3-*O*-rhamnosylrutinoside and isorhamnetin-3-*O*-rhamnosylrobinobiside showed less activity than the previous two compounds due to the presence of the additional compounds of the rhamnose group, which decreased its binding affinity. Similarly, for other compounds, rutinoside was replaced by the galactose moiety, which reduces their activity. Thus, it was concluded that the presence of the rutinoside moiety at the third position of the C ring and the absence of the *O*-methyl group at the B ring in the structure of flavonol are responsible for the higher binding stability [115]. Some of the recent in silico-based approaches for counteracting SARS-CoV-2 are shown in Table 1.

The structure–activity association study of flavones such as apigenin, luteolin, and quercetin has played a crucial role in SARS-CoV 3CLpro inhibition by substituting the hydroxyl group at C-30 in luteolin and quercetin [20]. A recent finding supports that luteolin establishes five hydrogen bonds with Gln189, Leu4, Asn142, Thr26, and the hydrophobic interaction with Met49 or Val3, in accordance with its lower binding energy [116].

Another study showing the inhibition of RNA viral replication suggested that RdRp can be a potential candidate for targeted drug production, which represents an important area for investigation. A molecular screening revealed that theaflavin can bind to the catalytic pocket of SARS-CoV-2 RdRp, resulting in a binding energy of −8.8 kcal/mol. A molecular test revealed that theaflavin would interact with SARS-CoV-2 RdRp’s catalytic pocket, showing −8.8 kcal/mol. According to molecular docking studies, the hydrophobic interactions are involved in the binding of theaflavin to RdRp. Furthermore, the hydrogen bonds were created between the functional theaflavin moieties and the Asp452, Arg553, and Arg724 residues of RdRp [117].

Thus, flavonoids can be potential anti-COVID-19 agents because they act as prospective inhibitors of the main protease (M^pro^) and other docking sites of the COVID-19 virus. Moreover, most of the compounds showed lower binding energy than presently used drugs and satisfied the studied parameters required for making a drug. In order to establish whether these flavonoids can be used as potential inhibitors of COVID-19, further in vitro and in vivo tests are needed before conducting clinical trials. 

## 5. Conclusions 

The study of COVID-19 treatments faces a significant obstacle for researchers due to increasing disease transmission and the impossibility of randomization. These life-threatening clinical trials are risky and have ethical issues. Some anti-viral drugs are given to patients, but alternative treatment options must be rapidly investigated and tested. Thus, in silico studies area powerful technique to screen probable antagonistic compounds such as flavonoids that can target the binding sites of SARS-CoV viral proteins via complex molecular interactions in viral attachment and replication. Structurally significant binding sites, strongly conserved domains within RdRp, and protease 3CLpro will accomplish this goal. Additionally, flavonoids will surely be powerful candidates in inhibiting or blocking several virus-host protein pathways of SARS-CoV-2. The synergistic combination of flavonoids with conventional drugs would be of great value. Several flavonoids such asquercetin, fisetin, apigenin, kaemferol, myricetin, daidzein, etc., interact with the main receptor-binding domain (RBD) of the spike protein and prevent the spread to viral receptors, but showed best results with M^pro^ by disrupting the activation/dimerization of the proteases. Quercetin is an exceptional candidate for further in vitro or in vivo studies because it shows strong interaction with the M^pro^ protease of SARS-CoV-2 at Glu290 and Asp289 and the RBD of the spike protein through in silico studies. Further in vitro and in vivo studies are needed for more in-depth research in this area, in addition to clinical trials for the validation of the in-silico results. There is also a need for collaborative studies across disciplines to examine such potential flavonoid compounds and maximize the improvement of targeted delivery approaches. This could speed up the production of successful COVID-19 treatments, which could save countless lives and respond expeditiously to potential future pandemics. We hope these collaborative studies and discussions will help scientists worldwide find flavonoids as potential candidates for the inhibition of coronavirus. 

## Figures and Tables

**Table 1:** Recent in silico studies of flavonoids against COVID-19.

Methods	No. of Compounds Tested	Structure (PDB ID)	Description	Binding Affinity for Molecules (Kcal/mol)	Results	References
SWISS DOCK	35	6LU7	Anti-viral drugs and 35 compounds were screened against chymotyripsin-like protease (3CLpro). UCSF chimera was used to visualize the interaction (hydrophobic bonds and H-bonds) between ligands and the amino acid of the targeted protein. ProTox was used to evaluate toxicity.	Cordifolin: −8.77 Anisofolin A: −8.72 Apigenin-7-glycoside: −8.36 Luteolin: −8.35 Laballenic acid: −8.13 Quercetin: −8.04 Luteolin-4-glucoside: −7.87	Apigenin-7-glycoside, luteolin, quercetin, and luteolin-4-glucoside showed least binding energy, meaning higher binding affinity with amino acids of protease.	[118]
Auto Dock Vina	19	6LU7 (main protease (M^pro^)) 6VXX (spike glycoprotein)	Molecular docking approach was used to study inhibition of two COVID-19 proteins, i.e., main protease (M^pro^) and spike glycoprotein by bioactive compounds. Lipinski’s rule of five was used to determine the efficacy of compounds as potential drugs. Nelfinavir, chloroquine, and hydroxyl chloroquine sulfate drugs were used as positive controls.	Hesperidin: −10.4 (spike glycoprotein), −8.3 (main protease) Nabiximols: −10.2 (spike glycoprotein), −8.0 (main protease) Pectolinarin: −9.8 (spike glycoprotein), −8.2 (main protease) Epigallocatechin gallate: −9.8 (spike glycoprotein), −7.8 (main protease) Rhoifolin: −9.5 (spike glycoprotein), 8.2 (main protease)	Hesperidine, cannabinoids, pectolinarin, epigallocatechin gallate. and rhoifolin showed better inhibitory activity for spike glycoprotein than control drugs. Hesperidine, cannabinoids, pectolinarin, and rhoifolin showed better inhibitory activity for main protease (M^pro^) than chloroquine and hydroxychloroquine sulfate drugs and almost similar to nelfinavir. However, hesperidine, pectolinarin, and rhoifolindonot follow Lipinski’s rule. Low bioavailability of some compounds, i.e., hesperidine, cannabinoids, and rhoifolin, may pose a problem during drug design.	[119]
MOE2010	--	6LU7 (main protease (M^pro^)) 6VW1 (PD-ACE2) 6LXT (RBD-S)	Compounds evaluated: (1) Curcuminoids from *Curcuma* sp. (2) Methoxy flavonoids from *Citrus* sp. (3) Phenolic compounds from *Caesalpinia sappan*. (4) Phenylpropanoid compounds from *Alpinia galanga*. Three protein targets were selected: (1) Receptor binding domain of spike protein (RBD-S). (2) Angiotension converting enzyme-2 receptor at protease domain (PD-ACE2). (3) Main protease (M^pro^). Docking score was used to evaluate binding affinity.	Curcumin: −11.82 (main protease), −8.39 (spike glycoprotein), −9.04 (RBD-ACE2) Hesperitin: −12.36 (main protease), −9.08 (spike glycoprotein), −6.72 (RBD-ACE2) Hesperidin: −13.51 (main protease), −9.61 (spike glycoprotein), −9.50 (RBD-ACE2) Naringenin: −12.44 (main protease), −7.40 (spike glycoprotein), −7.69 (RBD-ACE2) Brazilin: −12.36 (main protease), −7.50 (spike glycoprotein), −7.49 (RBD-ACE2) Galangin: −12.96 (main protease), −7.89 (spike glycoprotein), −7.60 (RBD-ACE2)	Hesperidin showed lowest binding energy for all three protein targets, i.e., −13.51 (main protease), −9.61 (RBD-S) and −9.50 (PD-ACE2). Docking score of hesperidin is less than lopinavir, meaning better interaction with protein targets. Other compounds also showed good affinity compared to reference compounds, but less than hesperidin. Citrus compounds showed better potential in inhibiting the development of COVID-19 followed by *Alpinia galangal*, *Caesalpinia sappan*, and *Curcuma*.	[120]
ClusPro (docking between spike protein fragment and human ACE2 receptor) SWISS DOCK (between compounds and the bound structure of the spike protein fragment and human ACE2 receptor)	5		Herperidin, emodin, anthroquinone, rhein, and chrysin phytochemicals were used in this study.	Hesperidin: −8.99 Emodin: −6.19 Anthroquinone: −6.15 Rhein: −8.73 Chrysin: −6.87	Hesperidin, emodin, and chrysin are considered as potential candidates to treat COVID-19. Hesperidin binds with the amino acids of the H1 and H2 helix of the ACE2 receptor protein. Emodin binds with the amino acids of the H2 helix of the ACE2 receptor protein. Anthroquinone and rhein are not considered as therapeutic agents against COVID-19 because of no interactions. Chrysin interacts with the amino acids of the H5 helix of the ACE2 receptor protein.	[121]
Autodock 4.2	13	6LU7	Docking score and binding energy were used to evaluate binding affinity. Lipinski’s rule of five was used to determine the efficacy of compounds as potential drugs.	Kaempferol: −8.58 Quercitin: −8.47 Luteolin-7-glucoside: −8.17 Demethoxycurcumin: −7.99 Naringenin: −7.89 Apigenin-7-glucoside: −7.83 Oleuropein: −7.31 Curcumin: −7.05 Catechin: −7.24 Epicatechin-gallate: −6.67	M^pro^ of COVID-19 shares 96% similarity with M^pro^ of SARS-CoV. Order of inhibition potential of selected compounds: nelfinavir > lopanavir > kaemferol > quercetin > luteolin-7-glucoside > demethoxycurcumin > naringenin > apigenin-7-glucoside > oleuropein > curcumin > catechin > epigallocatechin > zinger > allicin. All the compounds follow Lipinski’s rule of five.	[22]
Auto Dock Vina	72	6LU7	3CL ^pro^ was used as the active site for docking. Binding energy was calculated for checking binding affinity. ADME and toxicology of flavonoids were also performed.	Amentoflavone: −9.0 Gallocatechingallate: −8.3 Diosmin: −9 Epigallocatechin gallate: −8.3 Hidrosmin: −8.9 Catechingallate: −8.4 Elsamitrucin: −8.3 Pectolinaren: −8.3 Silibinin: −8.1 Oriemtim: −8.0 Isoquercetin= −8.0	All the flavonoids except auraptene have binding energy <−6 kcal/mol. Top ten flavonoids with lowest binding energy. The majority of flavonoids also had high predicted probabilities of being toxic to fathead minnows (FHM), honey bees (HBT), and Tetrahymena pyriformis (TPT), which should not be a concern for humans as they are all commonly consumed flavonoids.	[23]
Auto Dock Vina	14	6LU7	M^pro^ was used as the active site for docking. Polar H-bond was added to M^pro^ before docking followed by the addition of Kollman charges. Pymol 4.3.0, Ligplot+, and protein–ligand interaction profiler was used to analyze docking results.	Hesperidin Rutin Diosmin Apiin Diacetylcurcumin	Procyanidin b2 and mangiferin showed highest binding affinity with M^pro^ with binding energy of −9.4 Kcal/mol and −8.5 Kcal/mol, respectively. Azithromycin, an antibiotic, showed lowest binding energy, i.e., −13.4 kcal/mol. Both flavonoids form multiple H-bonds with the main chain of the residue in the substrate binding pocket, which inhibits the binding site of the inhibitor. Both flavonoids have binding affinity greater than hydroxyquinone, flavipiravir, and ramdesivir.	[122]
Swiss Dock	18	6LU7	18 compounds were extracted from 11 different species. Main focus was given to compounds that possess anti-malarial or anti-viral activity. Lipophilicity (log P) and aqueous solubility (log S) were calculated using ALOGPS 2.1 program.	Nictoflorin: −9.18 Astragalin: −8.68 Lupeol: −8.28 Aloenin: −9.13 Aloesin: −8.79 Berberine: −8.67 Sitosterol: −8.42 Curcumin: −8.44	Harsingar, Aloe vera, and giloy herbal plant compounds showed maximum affinity to M^pro^ of COVID-19. Nictofloein (−9.18 kcal/mol), astragalin (−8.68 kcal/mol), and lupeol (−8.28 kcal/mol) were extracted from harsingar; aloenin (−9.13 kcal/mol) and aloesin (−8.79 kcal/mol) were extracted from Aloe Vera; berbirine (−8.67 kcal/mol) and sitosterol (−8.42 kcal/mol) were extracted from giloy. Most compounds have log-P values in the range of 2.64–4.95. Lupeol, sitosterol, ursolic acid, and cannabidiol have log-P in the range of 5.12–7.27, which means they possess high hydrophobicity and poor absorption, whereas nictoflowin, astragalin, aloenin, aloesin, and quercetin have log-P (0.05–1.81), which means high absorption. Most compounds’ log-S value was in the range of −1 to −5, which implies less bioavailability. Nictoflowin, astragalin, aloenin, aloesin, and quercetin were considered as more biologically potent compounds asprotease inhibitors, as well as having good bioavailability.	[123]
Auto Dock Vina	7173 purchasable drugs and 4574 unique compounds and their stereoisomers	2DUC	High-resolution apoenzyme stricture of SARS-CoV-2 M^pro^ was used as the template. MTiopen screen web service was used for screening compounds.	Diosmin: −10.1 Hesperidin: −10.1 MK-3207: −10.1 Venetoclax: −10.0 Dihydroergocristine: −9.8 Bolazine: −9.8 R428: −9.8 Ditercalinium: −9.8 Etoposide-phosphate: −9.8	Hesperidin and diosmin fit well in the docking site and block the active site of the virus. Hesperidin and its 38 different stereoisomeric forms all were among the top scores. Good inhibitor of SARS-CoV 3CLpro with an IC_50_ value of 8.3 µM, whereas some of the mild adverse reactions of these flavonoids were also reported, such as stomach pain and nausea,	[21]

## Data Availability

Not applicable.

## References

[1] WHO (2022). Coronavirus Disease (COVID-19). https://covid19.who.int/.

[2] WHO (2020). WHO Welcomes Preliminary Results about Dexamethasone Use in Treating Critically Ill COVID-19 Patients. World Health Organization, Geneva. https://www.who.int/news-room/detail/16-06-2020-who-welcomes-preliminary-results-about-dexamethasone-use-in-treating-critically-ill-covid-19-patients.

[3] Sarma P., Prajapat M., Avti P., Kaur H., Kumar S., Medhi B. (2020). Therapeutic options for the treatment of 2019-novel coronavirus: An evidence-based approach. Indian J. Pharmacol..

[4] Balachandar V., Mahalaxmi I., Kaavya J., Vivek G., Ajithkumar S., Arul N., Singaravelu G., Nachimuthu S.K., Devi S.M. (2020). COVID-19 Emerging protective measures. Eur. Rev. Med. Pharmacol. Sci..

[5] McConkey B.J., Sobolev V., Edelman M. (2002). The performance of current methods in ligand–protein docking. Curr. Sci..

[6] Langer T., Hoffmann R.D. (2001). Virtual screening an effective tool for lead structure discovery. Curr. Pharm. Des..

[7] Bajorath J. (2002). Integration of virtual and high-throughput screening. Nat. Rev. Drug Discov..

[8] Jorgensen W.L. (2004). The many roles of computation in drug discovery. Science.

[9] Kitchen D.B., Decornez H., Furr J.R., Bajorath J. (2004). Docking and scoring in virtual screening for drug discovery: Methods and applications. Nat. Rev. Drug Discov..

[10] Xu Z., Peng C., Shi Y., Zhu Z., Mu K., Wang X., Zhu W. (2020). Nelfinavir was predicted to be a potential inhibitor of 2019-nCov main protease by an integrative approach combining homology modelling, molecular docking and binding free energy calculation. BioRxiv.

[11] Russo G.L., Tedesco I., Spagnuolo C., Russo M. (2017). Antioxidant polyphenols in cancer treatment: Friend, foe or foil?. Sem. Cancer Biolog..

[12] Spagnuolo C., Moccia S., Russo G.L. (2018). Anti-inflammatory effects of flavonoids in neurodegenerative disorders. Eur. J. Med. Chem..

[13] Lv Z., Chu Y., Wang Y. (2015). HIV protease inhibitors: A review of molecular selectivity and toxicity. HIV/AIDS.

[14] Lu H. (2020). Drug treatment options for the 2019-new coronavirus (2019-nCoV). Biosci. Trends.

[15] Jo S., Kim S., Shin D.H., Kim M.S. (2020). Inhibition of SARS-CoV 3CL protease by flavonoids. J. Enzyme.Inhib. Med. Chem..

[16] Hoffmann M., Kleine-Weber H., Schroeder S., Krüger N., Herrler T., Erichsen S., Schiergens T.S., Herrler G., Wu N.H., Nitsche A. (2020). SARS-CoV-2 cell entry depends on ACE2 and TMPRSS2 and is blocked by a clinically proven protease inhibitor. Cell.

[17] Sawikowska A. (2020). Meta-analysis of flavonoids with antiviral potential against coronavirus. Biom. Lett..

[18] Lin C.W., Tsai F.J., Tsai C.H., Lai C.C., Wan L., Ho T.Y., Hsieh C.C., Chao P.D.L. (2005). Anti-SARS coronavirus 3C-like protease effects of *Isatis indigotica* root and plant-derived phenolic compounds. Antivir. Res..

[19] Lau K.M., Lee K.M., Koon C.M., Cheung C.S.F., Lau C.P., Ho H.M., Lee M.Y.H., Au S.W.N., Cheng C.H.K., Bik-San Lau C. (2008). Immunomodulatory and anti-SARS activities of *Houttuyniacordata*. J. Ethnopharmacol..

[20] Ryu Y.B., Jeong H.J., Kim J.H., Kim Y.M., Park J.Y., Kim D., Naguyen T.T.H., Park S.J., Chang J.S., Park K.H. (2010). Biflavonoids from *Torreyan ucifera* displaying SARS-CoV 3CLpro inhibition. Bioorg. Med. Chem..

[21] Chen Y.W., Yiu C.P.B., Wong K.Y. (2020). Prediction of the SARS-CoV-2 (2019-nCoV) 3C-like protease (3CL pro) structure: Virtual screening reveals velpatasvir, ledipasvir, and other drug repurposing candidates. F1000Research.

[22] Khaerunnisa S., Kurniawan H., Awaluddin R., Suhartati S., Soetjipto S. (2020). Potential inhibitor of COVID-19 main protease (M^pro^) from several medicinal plant compounds by molecular docking study. Preprints.

[23] Peterson L. (2020). COVID-19 and Flavonoids: In silico Molecular Dynamics Docking to the Active Catalytic Site of SARS-CoV and SARS-CoV-2 Main Protease. https://papers.ssrn.com/sol3/papers.cfm?abstract_id=3599426.

[24] Adem S., Eyupoglu V., Sarfraz I., Rasul A., Ali M. (2020). Identification of potent COVID-19 main protease (M^pro^) inhibitors from natural polyphenols: An in silico strategy unveils a hope against corona. Preprints.

[25] Manjunath S.H., Thimmulappa R.K. (2021). Antiviral, immunomodulatory, and anticoagulant effects of quercetin and its derivatives: Potential role in prevention and management of COVID-19. J. Pharm. Anal..

[26] Khan A., Heng W., Wang Y., Qiu J., Wei X., Peng S., Saleem S., Khan M., Ali S.S., Wei D.Q. (2021). In silico and in vitro evaluation of kaempferol as a potential inhibitor of the SARS-CoV-2 main protease (3CLpro). Phytother. Res..

[27] Nguyen T.T.H., Jung J.H., Kim M.K., Lim S., Choi J.M., Chung B., Kim D.W., Kim D. (2021). The inhibitory effects of plant derivate polyphenols on the main protease of SARS coronavirus 2 and their structure–activity relationship. Molecules.

[28] Cherrak S.A., Merzouk H., Mokhtari-Soulimane N. (2020). Potential bioactive glycosylated flavonoids as SARS-CoV-2 main protease inhibitors: A molecular docking and simulation studies. PLoS ONE.

[29] Abd El-Mordy F.M., El-Hamouly M.M., Ibrahim M.T., Abd El-Rheem G., Aly O.M., Abd El-kader A.M., Youssif K.A., Abdelmohsen U.R. (2020). Inhibition of SARS-CoV-2 main protease by phenolic compounds from *Manilkarahexandra* (Roxb.) Dubard assisted by metabolite profiling and in silico virtual screening. RSC Adv..

[30] Santana F.P.R., Thevenard F., Gomes K.S., Taguchi L., Câmara N.O.S., Stilhano R.S., Ureshino R.P., Prado C.M., Lago J.H.G. (2021). New perspectives on natural flavonoids on COVID-19-induced lung injuries. Phytother. Res..

[31] Jo S., Kim S., Kim D.Y., Kim M.S., Shin D.H. (2020). Flavonoids with inhibitory activity against SARS-CoV-2 3CLpro. J. Enzyme Inhib. Med. Chem..

[32] Perlman S., Netland J. (2020). Coronaviruses post-SARS: Update on replication and pathogenesis. Nat. Rev.Microbiol..

[33] Gorbalenya A.E., Baker S.C., Baric R.S., de Groot R.J., Drosten C., Gulyaeva A.A., Haagmans B.L., Lauber C., Leontovich A.M., Neuman B.W. (2020). The species severe acute respiratory syndrome related coronavirus: Classifying 2019-nCoV and naming it SARS-CoV-2. Nat. Microbiol..

[34] Peiris J.S.M., Lai S.T., Poon L.L.M., Guan Y., Yam L.Y.C., Lim W., Nicholls J., Yee W.K.S., Yan W.W., Cheung M.T. (2003). Coronavirus as a possible cause of severe acute respiratory syndrome. Lancet.

[35] Li F. (2016). Structure, function, and evolution of coronavirus spike proteins. Ann. Rev. Virol..

[36] Lu R., Zhao X., Li J., Niu P., Yang B., Wu H., Wang W., Song H., Huang B., Zhu N. (2020). Genomic characterization and epidemiology of 2019 novel coronavirus: Implications for virus origins and receptor binding. Lancet.

[37] Cascella M., Rajnik M., Cuomo A., Dulebohn S.C., Di Napoli R. (2020). Features, Evaluation and Treatment Coronavirus (COVID-19). Statpearls [Internet].

[38] Guan W.J., Ni Z.Y., Hu Y., Liang W.H., Ou C.Q., He J.X., Liu L., Shan H., Lei C.L., Hui D.S. (2020). Clinical characteristics of coronavirus disease 2019 in China. N. Engl. J. Med..

[39] Yin Y., Wunderink R.G. (2018). MERS, SARS and other coronaviruses as causes of pneumonia. Respirology.

[40] Gorbalenya A.E., Baker S.C., Baric R., Groot R.J.D., Drosten C., Gulyaeva A.A., Haagmans B.L., Lauber C., Leontovich A.M., Neuman B.W. (2020). Severe acute respiratory syndrome-related coronavirus: The species and its viruses—A statement of the Coronavirus Study Group. BioRxiv.

[41] Zhang L., Lin D., Sun X., Curth U., Drosten C., Sauerhering L., Becker S., Rox K., Hilgenfeld R. (2020). Crystal structure of SARS-CoV-2 main protease provides a basis for design of improved α-ketoamide inhibitors. Science.

[42] Tai W., He L., Zhang X., Pu J., Voronin D., Jiang S., Zhou Y., Du L. (2020). Characterization of the receptor-binding domain (RBD) of 2019 novel coronavirus: Implication for development of RBD protein as a viral attachment inhibitor and vaccine. Cell Mol. Immunol..

[43] Zhong N.S., Zheng B.J., Li Y.M., Poon L.L.M., Xie Z.H., Chan K.H., Li P.H., Tan S.Y., Chang Q., Xie J.P. (2003). Epidemiology and cause of severe acute respiratory syndrome (SARS) in Guangdong, People’s Republic of China, in February, 2003. Lancet.

[44] Zhou P., Yang X.L., Wang X.G., Hu B., Zhang L., Zhang W., Si H.R., Zhu Y., Li B., Huang C.L. (2020). A pneumonia outbreak associated with a new coronavirus of probable bat origin. Nature.

[45] Peng X., Xu X., Li Y., Cheng L., Zhou X., Ren B. (2020). Transmission routes of 2019-nCoV and controls in dental practice. Int. J. Oral Sci..

[46] Guo Y.R., Cao Q.D., Hong Z.S., Tan Y.Y., Chen S.D., Jin H.J., Tan K.S., Wang D.Y., Yan Y. (2020). The origin, transmission and clinical therapies on coronavirus disease 2019 (COVID-19) outbreak—An update on the status. Military Med. Res..

[47] Chen Y., Liu Q., Guo D. (2020). Emerging coronaviruses: Genome structure, replication, and pathogenesis. J. Med. Virol..

[48] Prajapat M., Sarma P., Shekhar N., Avti P., Sinha S., Kaur H., Kumar S., Bhattacharyya A., Kumar H., Bansal S. (2020). Drug targets for corona virus: A systematic review. Indian J. Pharmacol..

[49] Sardar R., Satish D., Birla S., Gupta D. (2020). Comparative analyses of SAR-CoV2 genomes from different geographical locations and other coronavirus family genomes reveals unique features potentially consequential to host-virus interaction and pathogenesis. BioRxiv.

[50] Schoeman D., Fielding B.C. (2019). Coronavirus envelope protein: Current knowledge. Virol. J..

[51] Lai M.M., Cavanagh D. (1997). The Molecular Biology of Coronaviruses. Advances in Virus Research.

[52] Klausegger A., Strobl B., Regl G., Kaser A., Luytjes W., Vlasak R. (1999). Identification of a coronavirus hemagglutinin-esterase with a substrate specificity different from those of influenza C virus and bovine coronavirus. J. Virol..

[53] McBride R., Van Zyl M., Fielding B.C. (2014). The coronavirus nucleocapsid is a multifunctional protein. Viruses.

[54] Zumla A., Chan J.F., Azhar E.I., Hui D.S., Yuen K.Y. (2016). Coronaviruses—drug discovery and therapeutic options. Nat. Rev. Drug Discov..

[55] Jiang S., Shi Z., Shu Y., Song J., Gao G.F., Tan W., Guo D. (2020). A distinct name is needed for the new coronavirus. Lancet.

[56] Liu Z., Xiao X., Wei X., Li J., Yang J., Tan H., Zhu J., Zhang Q., Wum J., Liu L. (2020). Composition and divergence of coronavirus spike proteins and host ACE2 receptors predict potential intermediate hosts of SARS-CoV-2. J. Med. Virol..

[57] Phan T. (2020). Novel coronavirus: From discovery to clinical diagnostics. Infect. Genet. Evol..

[58] Zhao Y., Zhao Z., Wang Y., Zhou Y., Ma Y., Zuo W. (2020). Single-cell RNA expression profiling of ACE2, the putative receptor of Wuhan 2019-nCov. BioRxiv.

[59] Jafary F., Jafari S., Ganjalikhany M.R. (2021). In silico investigation of critical binding pattern in SARS-CoV-2 spike protein with angiotensin-converting enzyme 2. Sci. Rep..

[60] Ou X., Lium Y., Lei X., Li P., Mi D., Ren L., Guo L., Guo R., Chen T., Hu J. (2020). Characterization of spike glycoprotein of SARS-CoV-2 on virus entry and its immune cross-reactivity with SARS-CoV. Nat. Commun..

[61] Grottesi A., Besker N., Emerson A., Manelfi C., Beccari A.R., Frigerio F., Lindahl E., Cerchia C., Talarico C. (2020). Computational studies of SARS-CoV-2 3CLpro: Insights from MD simulations. Int. J. Mol. Sci..

[62] Satarker S., Nampoothiri M. (2020). structural proteins in severe acute respiratory syndrome coronavirus-2. Arch. Med. Res..

[63] Xu C., Wang Y., Liu C., Zhang C., Han W., Hong X., Wang Y., Hong Q., Wang S., Zhao Q. (2021). Conformational dynamics of SARS-CoV-2 trimeric spike glycoprotein in complex with receptor ACE2 revealed by cryo-EM. Sci. Adv..

[64] Gralinski L.E., Menachery V.D. (2020). Return of the Coronavirus: 2019-nCoV. Viruses.

[65] Walker L.M., Burton D.R. (2018). Passive immunotherapy of viral infections:’ Super-antibodies’ enter the fray. Nat. Rev.Immunol..

[66] Astuti I. (2020). Severe acute respiratory syndrome coronavirus 2 (SARS-CoV-2): An overview of viral structure and host response. Diabetes Metab. Syndr. Clin. Res. Rev..

[67] Casadevall A., Pirofski L.A. (2020). The convalescent sera option for containing COVID-19. J. Clin. Investig..

[68] Kruse R.L. (2020). Therapeutic strategies in an outbreak scenario to treat the novel coronavirus originating in Wuhan, China. F1000Research.

[69] Shetty R., Ghosh A., Honavar S.G., Khamar P., Sethu S. (2020). Therapeutic opportunities to manage COVID-19/SARS-CoV-2 infection: Present and future. Indian J. Ophthalmol..

[70] Ho T.Y., Wu S.L., Chen J.C., Li C.C., Hsiang C.Y. (2007). Emodin blocks the SARS coronavirus spike protein and angiotensin-converting enzyme 2 interaction. Antivir. Res..

[71] Smith M., Smith J.C. (2020). Repurposing therapeutics for COVID-19: Supercomputer-based docking to the SARS-CoV-2 viral spike protein and viral spike protein-human ACE2 interface. ChemRxiv.

[72] Liu C., Zhou Q., Li Y., Garner L.V., Watkins S.P., Carter L.J., Smoot J., Gregg A.C., Daniels A.D., Jervey S. (2020). Research and development on therapeutic agents and vaccines for COVID-19 and related human coronavirus diseases. ACS Cent. Sci..

[73] Matsuyama S., Nao N., Shirato K., Kawase M., Saito S., Takayama I., Nagata N., Sekizuka T., Katoh H., Kato F. (2020). Enhanced isolation of SARS-CoV-2 by TMPRSS2-expressing cells. Proc. Nat. Acad. Sci. USA.

[74] Yamamoto N., Yang R., Yoshinakam Y., Amari S., Nakano T., Cinatl J., Rabenau H., Doerr H.W., Hunsmann G., Otaka A. (2004). HIV protease inhibitor nelfinavir inhibits replication of SARS-associated coronavirus. Biochem. Biophys. Res. Commun..

[75] Park J.Y., Jeong H.J., Kim J.H., Kim Y.M., Park S.J., Kim D., Park K.H., Lee W.S., Ryu Y.B. (2012). Diarylheptanoids from *Alnus japonica* inhibit papain-like protease of severe acute respiratory syndrome coronavirus. Biol. Pharm. Bull..

[76] Teijaro J.R. (2016). Type I interferons in viral control and immune regulation. Curr. Opin. Virol..

[77] Sathish J.G., Sethum S., Bielsky M.C., De Haan L., French N.S., Govindappa K., Green J., Griffiths C.E., Holgat S., Jones D. (2013). Challenges and approaches for the development of safer immunomodulatory biologics. Nat. Rev. Drug Discov..

[78] Gong J., Xu W., Zhang J. (2007). Structure and functions of influenza virus neuraminidase. Curr. Med. Chem..

[79] McAuley J.L., Gilbertson B.P., Trifkovic S., Brown L.E., McKimm-Breschkin J.L. (2019). Influenza virus neuraminidase structure and functions. Front. Microbiol..

[80] Ganjhu R.K., Mudgal P.P., Maity H., Dowarha D., Devadiga S., Nag S., Arun kumar G. (2015). Herbal plants and plant preparations as remedial approach for viral diseases. Virus Dis..

[81] Solanki I., Parihar P., Mansuri M.L., Parihar M.S. (2015). Flavonoid-based therapies in the early management of neurodegenerative diseases. Adv. Nutr..

[82] Oh J.S., Kim H., Vijayakumar A., Kwon O., Kim Y., Chang N. (2017). Association of dietary flavonoid intake with prevalence of type 2 diabetes mellitus and cardiovascular disease risk factors in Korean women aged≥ 30 years. J. Nutri. Sci. Vitaminol..

[83] Dalgaard F., Bondonno N.P., Murray K., Bondonno C.P., Lewis J.R., Croft K.D., Kyro C., Gislason G., Scalbert A., Cassidy A. (2019). Associations between habitual flavonoid intake and hospital admissions for atherosclerotic cardiovascular disease: A prospective cohort study. Lancet Planet Health.

[84] McCullough M.L., Peterson J.J., Patel R., Jacques P.F., Shah R., Dwyer J.T. (2012). Flavonoid intake and cardiovascular disease mortality in a prospective cohort of US adults. Am. J. Clin. Nutr..

[85] Kim Y., Je Y. (2017). Flavonoid intake and mortality from cardiovascular disease and all causes: A meta-analysis of prospective cohort studies. Clin. Nutr. ESPEN.

[86] Zhou Z., Luo B., Liu X., Chen M., Lan W., Iovanna J.L., Peng L., Xia Y. (2019). Flavonoid–alkylphospholipid conjugates elicit dual inhibition of cancer cell growth and lipid accumulation. Chem. Commun..

[87] Loung C.Y., Fernando W., Rupasinghe H.P., Hoskin D.W. (2019). Apple peel flavonoid fraction 4 suppresses breast cancer cell growth by cytostatic and cytotoxic mechanisms. Molecules.

[88] Navarra M., Femia A.P., Romagnoli A., Tortora K., Luceri C., Cirmi S., Ferlazzo N., Caderni G. (2019). A flavonoid-rich extract from bergamot juice prevents carcinogenesis in a genetic model of colorectal cancer, the Pirc rat (F344/NTac-Apc am1137). Eur. J. Nutr..

[89] Wei F., Ma S.C., Ma L.Y., But P.P.H., Lin R.C., Khan I.A. (2004). Antiviral Flavonoids from the Seeds of *Aesculus chinensis*. J. Nat. Prod..

[90] Téllez M.A., Téllez A.N., Vélez F., Ulloa J.C. (2015). In vitro antiviral activity against rotavirus and astrovirus infection exerted by substances obtained from *Achyrocline bogotensis* (Kunth) DC (Compositae). BMC Complement Altern. Med..

[91] Lopes B.R.P., da Costa M.F., Ribeiro A.G., da Silva T.F., Lima C.S., Caruso I.P., de Araujo G.C., Kubo L.H., Iacovelli F., Falconi M. (2020). Quercetin pentaacetate inhibits in vitro human respiratory syncytial virus adhesion. Virus Res..

[92] Badshah S.L., Faisal S., Muhammad A., Poulson B.G., Emwas A.H., Jaremko M. (2021). Antiviral activities of flavonoids. Biomed. Pharmacother..

[93] Russo M., Moccia S., Spagnuolo C., Tedesco I., Russo G.L. (2020). Roles of flavonoids against coronavirus infection. Chem. Biol. Interact..

[94] Chen F., Chan K.H., Jiang Y., Kao R.Y.T., Lu H.T., Fan K.W., Cheng V.C., Tsui W.H., Hung I.F., Lee T.S. (2004). In vitro susceptibility of 10 clinical isolates of SARS coronavirus to selected antiviral compounds. J. Clin. Virol..

[95] Yi L., Li Z., Yuan K., Qu X., Chen J., Wang G., Zhang H., Lou H., Zhu L., Jiang P. (2004). Small molecules blocking the entry of severe acute respiratory syndrome coronavirus into host cells. J. Virol..

[96] Park J.Y., Yuk H.J., Ryu H.W., Lim S.H., Kim K.S., Park K.H., Ryu Y.B., Lee W.S. (2017). Evaluation of polyphenols from *Broussonetiapapyrifera* as coronavirus protease inhibitors. J. Enzy. Inhibit. Med. Chem..

[97] Jo S., Kim H., Kim S., Shin D.H., Kim M.S. (2019). Characteristics of flavonoids as potent MERS-CoV 3C-like protease inhibitors. Chem. Biol. Drug Design..

[98] Roh C. (2012). A facile inhibitor screening of SARS coronavirus N protein using nanoparticle-based RNA oligonucleotide. Int. J. Nanomed..

[99] Pandey P., Rane J.S., Chatterjee A., Kumar A., Khan R., Prakash A., Ray S. (2020). Targeting SARS-CoV-2 spike protein of COVID-19 with naturally occurring phytochemicals: An in silico study for drug development. J. Biomol. Struct. Dyn..

[100] Stoermer M. (2020). Homology Models of Coronavirus 2019-nCoV 3CLpro Protease. Chemrxiv.

[101] WFMJ (2020). COVID-19 and Flavonoids: Study Examines How Infection Can Be Minimized. https://www.wfmj.com/story/42088783/covid-19-and-flavonoids-study-examines-how-infection-can-be-minimized.

[102] Nguyen T.T.H., Woo H.J., Kang H.K., Kim Y.M., Kim D.W., Ahn S.A., Xia Y., Kim D. (2012). Flavonoid-mediated inhibition of SARS coronavirus 3C-like protease expressed in *Pichia pastoris*. Biotechnol. Lett..

[103] Schwarz S., Sauter D., Wang K., Zhang R., Sun B., Karioti A., Bilia A.R., Efferth T., Schwarz W. (2014). Kaempferol derivatives as antiviral drugs against the 3A channel protein of coronavirus. Planta Med..

[104] Boroujeni A.S., Sani M.R.M. (2021). Anti-inflammatory potential of Quercetin in COVID-19 treatment. J. Inflamm..

[105] Omrani M., Keshavarz M., NejadEbrahimi S., Mehrabi M., McGaw L.J., Ali Abdalla M., Mehrbod P. (2021). Potential natural products against respiratory viruses: A perspective to develop anti-COVID-19 medicines. Front. Pharmacol..

[106] Wang J. (2020). Fast identification of possible drug treatment of coronavirus disease-19 (COVID-19) through computational drug repurposing study. J. Chem. Inf. Model..

[107] Pandey P., Singhal D., Khan F., Arif M. (2021). An In Silico Screening on *Piper nigrum*, *Syzygium aromaticum* and *Zingiber officinale* roscoe derived compounds against SARS-CoV-2: A drug repurposing approach. Biointerface Res. Appl. Chem..

[108] Gogoi N., Chowdhury P., Goswami A.K., Das A., Chetia D., Gogoi B. (2021). Computational guided identification of a citrus flavonoid as potential inhibitor of SARS-CoV-2 main protease. Mol. Divers..

[109] Bhardwaj V.K., Singh R., Sharma J., Rajendran V., Purohit R., Kumar S. (2021). Identification of bioactive molecules from tea plant as SARS-CoV-2 main protease inhibitors. J. Biomol. Struc. Dyn..

[110] ColungaBiancatelli R.M.L., Berrill M., Marik P.E. (2020). Quercetin and vitamin C: An experimental, synergistic therapy for the prevention and treatment of SARS-CoV-2 related disease (COVID-19). Front. Immunol..

[111] Swain S.S., Singh S.R., Sahoo A., Hussain T., Pati S. (2021). Anti-HIV-drug and phyto-flavonoid combination against SARS-CoV-2: A molecular docking-simulation base assessment. J. Biomol. Struc. Dyn..

[112] Swain S.S., Paidesetty S.K., Dehury B., Das M., Vedithi S.C., Padhy R.N. (2020). Computer-aided synthesis of dapsone-phytochemical conjugates against dapsone-resistant *Mycobacterium leprae*. Sci. Rep..

[113] Vijayakumar B.G., Ramesh D., Joji A., Kannan T. (2020). In silico pharmacokinetic and molecular docking studies of natural flavonoids and synthetic indole chalcones against essential proteins of SARS-CoV-2. Eur. J. Pharmacol..

[114] Ngwa W., Kumar R., Thompson D., Lyerly W., Moore R., Reid T.E., Lowe H., Toyang N. (2020). potential of flavonoid-inspired phytomedicines against COVID-19. Molecules.

[115] Owis A.I., El-Hawary M.S., El Amir D., Aly O.M., Abdelmohsen U.R., Kamel M.S. (2020). Molecular docking reveals the potential of *Salvadorapersica* flavonoids to inhibit COVID-19 virus main protease. RSC Adv..

[116] Yu R., Chen L., Lan R., Shen R., Li P. (2020). Computational screening of antagonist against the SARS-CoV-2 (COVID-19) coronavirus by molecular docking. Int. J. Antimicrob. Agents..

[117] Lung J., Lin Y.S., Yang Y.H., Chou Y.L., Shu L.H., Cheng Y.C., Liu H.T., Wu C.Y. (2020). The potential chemical structure of anti-SARS-CoV-2 RNA-dependent RNA polymerase. J. Med. Virol..

[118] Mishra R.C., Kumari R., Yadav S., Yadav J.P. (2020). Antiviral potential of phytoligands against chymotrypsin-like protease of COVID-19 virus using molecular docking studies: An optimistic approach. Preprints.

[119] Tallei T.E., Tumilaar S.G., Niode N.J., Fatimawali F., Kepel B.J., Idroes R., Effendi Y. (2020). Potential of plant bioactive compounds as SARS-CoV-2 main protease (M^pro^) and spike (S) glycoprotein inhibitors: A molecular docking study. Preprints.

[120] Utomo R.Y., Meiyanto E. (2020). Revealing the potency of citrus and galangal constituents to halt SARS-CoV-2 infection. Preprints.

[121] Basu A., Sarkar A., Maulik U. (2020). Computational approach for the design of potential spike protein binding natural compounds in SARS-CoV2. Res. Sq..

[122] Chauhan A., Kalra S. (2020). Identification of potent COVID-19 main protease (M^pro^) inhibitors from flavonoids. Res. Sq..

[123] Srivastava A.K., Kumar A., Misra N. (2020). On the inhibition of COVID-19 protease by Indian herbal plants: An in silico investigation. arXiv.

